# Newborn screening for methylmalonic acidemia: insights from a retrospective analysis in Hefei, China

**DOI:** 10.1186/s13023-026-04225-5

**Published:** 2026-03-04

**Authors:** Yan Wang, Qingqing Ma, WeiDong Li, Yong Huang, Wangsheng Song, Hongyu Xu, Peng Zhu, Haili Hu

**Affiliations:** 1 Anhui Women and Children’s Medical Center (Hefei Women and Children’s Health Center), Hefei, Anhui 230001 China; 2 Department of Maternal, Child and Adolescent Health, School of Public Health, Anhui Medical University, Hefei, 230022 China

**Keywords:** Methylmalonic acidemia, Newborn screening, *MMACHC*, MS/MS

## Abstract

**Background:**

Methylmalonic acidemia (MMA) is an autosomal recessive inheritedcongenital metabolic enzyme deficiency disorder that can lead to multi-systemdamage, including the nervous, hematopoietic, hepatic, and renal systems, causingsevere symptoms in the neonatal period and long-term nutritional issues. The detection rate of MMA exhibits significant regional variations. This study aims to find out the incidence rate, biochemical and molecular characteristics, and follow-up status in the Hefei Neonatal Cohort in China.

**Results:**

From 2016 to 2023, 34 MMA cases were confirmed biochemically and genetically, comprising 25 combined-type (73.5%) and 9 isolated-type (26.5%) presentations. MMACHC mutations predominated (68.8%), with c.609G>A accounting for 45.5% of variants. Longitudinal surveillance of 26 MMA patients revealed significant morbidity, with 3 cases (11.5%) succumbing to disease complications during follow-up. Comprehensive assessments identified nutritional deficits in 6 patients (23.1%) and neurodevelopmental delays in 4 cases (15.4%), highlighting the multisystemic nature of MMA progression.

**Conclusion:**

This study provides a comprehensive characterization of the epidemiological, genetic, and clinical profiles of MMA in the Hefei neonatal cohort. Our results confirm a substantial burden of MMA in this Chinese population. The high morbidity and mortality observed, alongside significant nutritional and neurodevelopmental complications, underscore the multisystemic impact of MMA and highlight the critical need for early diagnosis, sustained follow-up, and genotype-tailored management strategies to improve long-term outcomes.

## Introduction

Methylmalonic acidemia (MMA) is a rare autosomal recessive disorder of organic acid metabolism [1]. The global prevalence of neonatal MMA is 1.14 per 100,000 live births, with higher rates observed in Asia and Africa [2–4]. Notably, Chinese epidemiological studies demonstrate significant geographical disparities, with Shandong Province reporting the highest prevalence (1/2,390-1/6,530), contrasting with lower rates in Zhejiang (1/66,000) and Shanghai (1/28,000) [5–7]. Patients with methylmalonic acidemia (MMA) have a high mortality rate and poor long-term survival prognosis, particularly those with the mut-type MMA. Although there has been some improvement in recent years, the mortality rate remains around 40% [8].

The pathogenesis of MMA primarily involves genetic mutations affecting methylmalonyl-CoA mutase (MCM) function or cobalamin (VitB12) metabolism, resulting in impaired conversion of methylmalonic acid to succinic acid [9, 10]. This metabolic blockade leads to pathological accumulation of methylmalonic acid, methylcitric acid, and 3-hydroxypropionic acid, subsequently causing multi-organ damage involving neurological, hepatic, renal, and gastrointestinal systems. The clinical presentation of MMA is remarkably heterogeneous, with non-specific symptoms occurring across all age groups, frequently leading to misdiagnosis or delayed diagnosis [7, 11]. Definitive diagnosis typically requires biochemical and genetic confirmation. The primary clinical management involves a protein-restricted diet or specialized formula devoid of branched-chain amino acids, supplemented with L-carnitine and cobalamin (for cobalamin-responsive patients) [1]. However, metabolic decompensation cannot be completely prevented, and neurological sequelae remain frequently observed.

Newborn screening utilizing tandem mass spectrometry has emerged as a critical strategy for early MMA detection, enabling timely intervention that significantly reduces mortality and morbidity associated with this condition [12, 13]. This comprehensive analysis aims to characterize the epidemiological profile, metabolic marker dynamics, and genetic mutation spectrum of MMA in Hefei region, thereby providing evidence-based recommendations for optimizing screening protocols and clinical management.

## Subjects and methods

### Study population and data sources

This study systematically analyzed the epidemiological characteristics, genotypic distribution, and clinical outcomes of methylmalonic acidemia (MMA) using a newborn screening cohort comprising 782,930 infants delivered at various maternity institutions in Hefei between January 2016 and December 2023. The newborn screening data and live birth statistics were obtained from the standardized monthly reporting system of Hefei districts, with regular quality control audits conducted by municipal-level expert panels to ensure data reliability.

### Initial screening of MMA using tandem mass spectrometry

The screening protocol strictly adhered to the “Technical Specifications for Newborn Screening Blood Collection (2010 Edition)” with standardized procedures: trained obstetric nurses collected heel blood samples at 72 h post-delivery (after adequate feeding) to prepare qualified dried blood spots (≥ 8.0 mm diameter with uniform bilateral penetration and no contamination), which were air-dried at room temperature, sealed, and stored at 2–8℃before cold-chain transportation to Hefei Newborn Screening Center Laboratory. Specimens were analyzed using a Waters Xevo TQD tandem mass spectrometry system (USA) with PerkinElmer NeoBase non-derivatized reagents to quantify including C3, C2, methionine, and others. The Hefei Newborn Screening Center is the sole institution responsible for newborn disease screening in Hefei City. Specimens collected from live births throughout the city were transported to the center’s laboratory via a cold-chain logistics system. All samples were analyzed in a single central laboratory, ensuring consistency in test results.

### Sanger sequencing for variant validation

For neonates with positive screening results, confirmatory genetic testing was systematically performed through an integrated molecular diagnostic protocol. Venous whole blood (3 mL) collected from probands and parents in EDTA-K2 anticoagulant tubes was processed at Beijing MyGenostics Medical Laboratory, where genomic DNA was extracted using Tiangen DP318-03 kits and stored at -20 °C. After capturing the target exons, next-generation sequencing (NGS) was performed. The obtained sequencing sequences were compared with the human genome hg19 reference sequence, and the coverage and sequencing quality of the target region were evaluated. Bioinformatics analysis and pathogenicity analysis of the variations were conducted. The target region included the coding sequence of the target gene and its adjacent intron region within ± 10 bp. Nucleotide variations with clear chromosomal locations were verified for the proband and the parents of the affected children using Sanger sequencing. All clinically significant variants were verified by Sanger sequencing and interpreted according to ACMG 2015 guidelines, with stringent quality control measures including internal positive controls, dual clinical geneticist review, and periodic NIST reference material calibration to ensure accurate identification of pathogenic variants in *MMUT*, *MMACHC* and related genes(The sequencing panel includes MMA-related genes: MMACHC, MMADHC, MMUT, MMAA, MMAB, MCEE, ABCD4, ACSF3, ALDH6A1, GPHN, HCFC1, LMBRD1, and SUCLG1). Variant pathogenicity is classified into five categories: pathogenic, likely pathogenic, variant of uncertain significance, likely benign, and benign. Each pathogenic criterion is weighted as very strong (PVS1), strong (PS1-PS4), moderate (PM1-PM6), or supporting (PP1-PP5), while each benign criterion is weighted as stand-alone (BA1), strong (BS1-BS4), or supporting (BP1-BP6). An allele frequency (AF) threshold of 1% is applied [14].

### Diagnostic classification of MMA

The definitive diagnosis of MMA requires integrated biochemical and genetic assessments. Combined MMA (with homocystinuria) is characterized by elevated C3/C2 ratio (with or without increased C3) and/or urinary methylmalonic acid accumulation, concomitant with hyperhomocysteinemia, and confirmed by pathogenic variants in cobalamin metabolism genes (*MMACHC*, *MMADHC*, *HCFC1*, *LMBRD1*, *ABCD4*, or *CD320*). Isolated MMA results from biallelic pathogenic variants affecting methylmalonyl-CoA mutase pathway components (*MMUT*, *MMAA*, *MMAB*, *MCEE*, *ACSF3*, *SUCLG1*, *SUCLA2*, or *ALDH6A1*).

### Birth weight assessment

Neonatal birth weight was classified according to the WHO Multicentre Growth Reference Study Group standards using gestational age-specific percentiles: small-for-gestational-age (SGA, < 10th percentile), appropriate-for-gestational-age (AGA, 10th-90th percentile), and large-for-gestational-age (LGA, > 90th percentile).

### Growth and developmental follow-up

Longitudinal follow-up incorporated standardized anthropometric and neurodevelopmental assessments according to WHO criteria. Physical growth parameters were evaluated through: (1) weight-for-age (underweight: <-2SD), (2) weight-for-height (wasting: <-2SD [moderate − 3SD to -2SD; severe <-3SD]; overweight: +1SD to + 2SD; obesity: ≥+2SD [moderate + 2SD to + 3SD; severe > + 3SD]), and (3) height-for-age (stunting: <-2SD). Neurodevelopmental progress was assessed using specialist evaluations from tertiary pediatric centers, with all measurements referenced to gender- and age-specific WHO growth standards (M: population mean; SD: standard deviation). This comprehensive monitoring protocol enabled systematic detection of MMA-associated growth deviations and timely nutritional interventions.

## Statistical analyses

This study employed a two-tiered analytical approach, combining initial tandem mass spectrometry (MS/MS) screening with subsequent genomic sequencing, to comprehensively characterize MMA-positive neonates. The dataset was analyzed both collectively and stratified by detection methodology. Categorical variables are presented as absolute counts with corresponding percentages (n, %). All statistical analyses were performed using the SPSS statistical package (version 17.0; IBM Corporation, Armonk, NY, USA).

## Results

### Diagnostic profile of MMA in Hefei’s neonatal screening

From 2016 to 2023, a total of 814,022 live births were recorded in Hefei City, of which 782,930 newborns underwent screening. The overall screening rate was 96.18%, with the annual screening rate reaching a peak of 99.38% during this period. Screening identified 34 cases of MMA, corresponding to an incidence rate of 1 in 23,027(Suspected cases that died before diagnosis were excluded from the calculation of incidence rates).

**Fig. 1:**
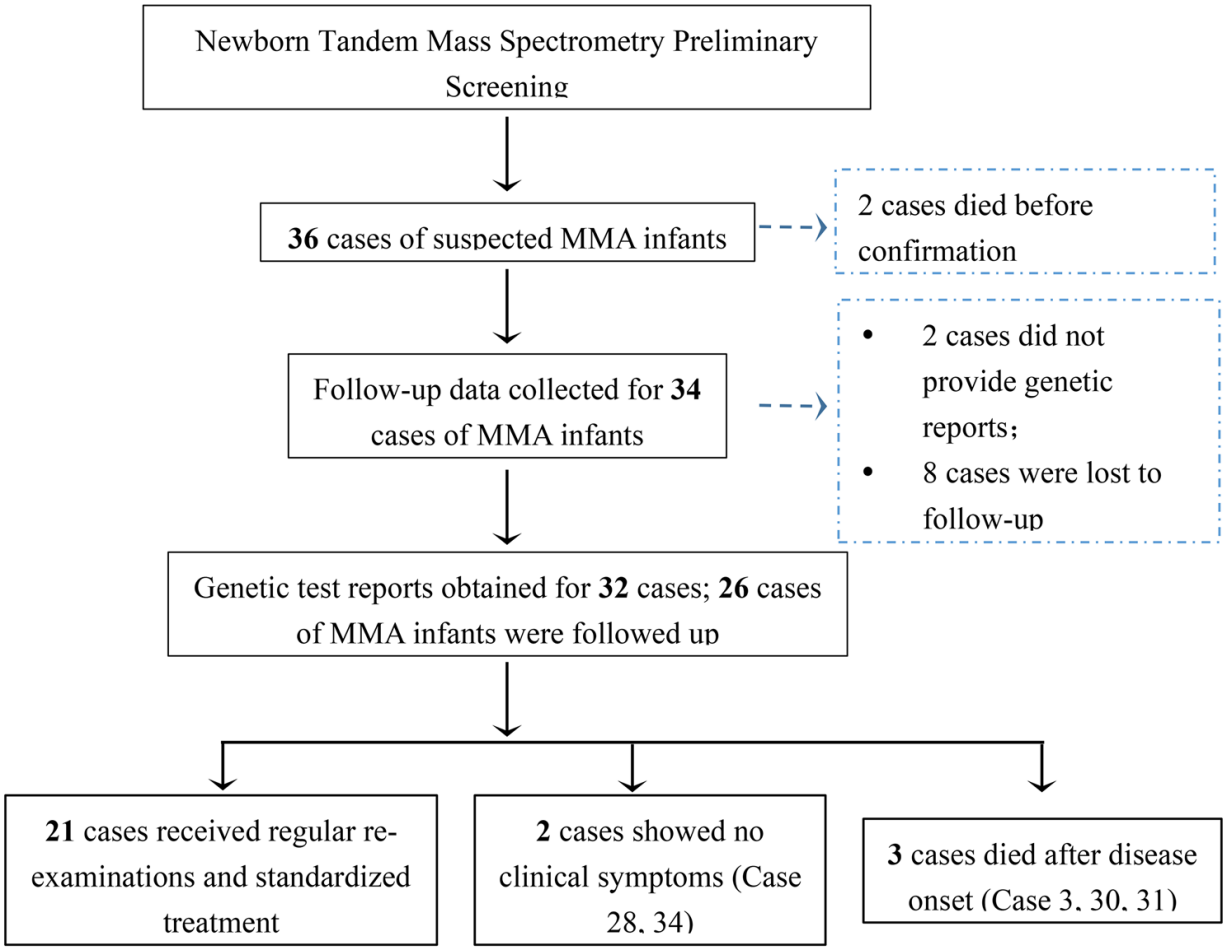
Study flowchart

MS/MS analysis identified 36 suspected MMA cases based on elevated C3 and C3/C2 ratios, including two neonates who died from rapid disease progression before confirmatory testing as showed in Fig. 1. Genetic sequencing ultimately confirmed 34 MMA cases (male: female = 16:18), with clinical stratification revealing 25 combined-type cases (cobalamin metabolism gene mutations) and 9 isolated-type cases (7 vitamin B12-nonresponsive with MMUT mutations). The cohort included 3 preterm infants and showed distinct birthweight patterns (12 SGA, 1 LGA) (as showed in Table 1). There is no significant differences in gestational age or sex distribution were observed between the two patient groups in MMA subtypes (*P* > 0.05). Notably, the mean diagnostic age improved significantly from 54 days overall to 43 days in the most recent triennium (2021–2023), demonstrating enhanced screening efficiency.

**Table 1:** Clinical and demographic features of 34 confirmed cases

Variable	Subtype *n*(%)	
	Combined	Isolated
Gender		
Female	9(81.82)	2(18.18)
Male	16(69.57)	7(30.43)
Gestational age at birth		
Large for Gestational Age	0(0.00)	0(0.00)
Appropriate for Gestational Age	15(65.22)	8(34.78)
Small for Gestational Age	10(90.91)	1(9.09)
Clinical status		
Under treatment	15(78.95)	4(21.05)
Symptomatic with mortality	1(33.33)	2(66.67)
Asymptomatic (untreated)	3(75.00)	1(25.00)
Lost to follow-up	6(75.00)	2(25.00)
Suspected cases of death before diagnosis	2	

### MS/MS reveals biochemical characteristics in MMA patients

Biochemical characteristics analysis of methylmalonic acidemia (MMA) patients revealed significant differences between subtypes in tandem mass spectrometry (MS/MS) detection (Tables 2 and 3). During initial screening (*n* = 36), C3 and its derivatives demonstrated high sensitivity: 91.67% (33/36) of infants exhibited elevated C3 levels, while 100% (36/36) showed abnormal C3/C2 ratios. Methionine (Met) levels were reduced in 47.83% (11/23) of CblC-type patients. Three combined-type patients showed no homocysteine (HCY) abnormalities, while 1 isolated-type case presented with detectable abnormalities (MUT variant). Urinary organic acid analysis (*n* = 22) further confirmed methylmalonic acid (UMA) as the most stable biomarker (90.9% abnormality rate), followed by methylcitric acid (MCA, 59.1%) and 3-hydroxypropionic acid (HPA, 40.9%). All recalled cases were diagnostically confirmed as MMA, with 4 infants undergoing external validation. Follow-up data (*n* = 21) demonstrated that despite treatment, 33.3% of patients maintained C3 or C3/C2 abnormalities. Urinary organic acid analysis revealed stable abnormality rates of approximately 30% for UMA, MCA, and HPA, suggesting these markers may reflect chronic metabolic states of the disease.

**Table 2:** Tandem mass spectrometry screening and genetic mutation analysis of MMA cases

No.	Subtype	Primary Screening Results (Cutoff)			Recall Review Results (Cutoff)			Genes	Nucleotide Change	Protein Change	Zygosity
		C3 (0.2-4)	C3/C2 (0.03–0.2)	Met (7.23-40)	C3(0.2-4)	C3/C2(0.03–0.2)	Met(7.23-40)				
1	CblB	**17.24**	**0.723**	36.11	**15.73**	**2.43**	24.4	MMACHC	c.609G> A	p.L128P	Hom
2	CblC	**11.62**	**0.728**	**2.13**	**7.1**	**1.151**	8.7	MMACHC	c.656_658del/c.609G> A	p.219_220del/p.W203X	CH
3^c^	CblC	**16.24**	**0.589**	8.61	**6.6**	**1.049**	NA	MMACHC	c.609G> A/c.656_658del	p.W203X/p.219_220del	Ch
4	CblC	**13.38**	**0.605**	11.01	**10.22**	**2.256**	8.53	MMACHC	c.656_658del/c.609G> A	p.219_220del/p.W203X	CH
5	CblC	**5.81**	**0.254**	15.73	**4.38**	**0.425**	14.96	MMACHC	c.482G> A/c.609G> A	p.R161Q/p.W203X	CH
6	CblC	**8.93**	**0.325**	**5.89**	**4.93**	**0.818**	**5.77**	MMACHC	c.394 C> T/c.656_658del	p.R132X/p.219_220del	CH
7	CblC	**8.97**	**1.67**	**5.29**	**9.53**	**1.354**	10.76	MMACHC	c.609G> A/c.567dup	p.w203/p.I190Yfs*13	CH
8	CblC	**15.43**	**1.011**	**6.48**	**8.94**	**1.706**	**5.06**	MMACHC	c.609G> A	p.w203*	Hom
9	CblC	3.87	**0.949**	**3.47**	**4.5**	0.077	34.55	MMACHC	c.609G> A	p.W203*	Hom
10	CblC	**5.52**	**1.605**	**2.96**	NA^a^	NA^a^	NA^a^	MMACHC	c.658_660del/c.481 C> T	p.K220del/p.R161*	CH
11	CblC	**8.76**	**1.708**	**6.81**	NA^a^	NA^a^	NA^a^	MMACHC	c.609G> A/c.627T> C	p.W203*/ p.D209D	CH
12	CblC	**5.35**	**0.394**	18.31	3.76	**0.708**	27.2	MMACHC	c.609G> A/c.541G> T	p.w203*/p.D181Y	CH
13	CblC	**6.91**	**0.833**	**6.76**	**4.98**	**0.767**	8.74	MMACHC	c.1 A> G/c.315 C> G	p.M1?/p.W20	CH
14	CblC	**7.44**	**0.732**	**6.18**	**7.43**	**2.502**	**4.85**	MMACHC	c.2T> A/c.609G> A	p.M1?/p.W203	CH
15	CblC	**5.38**	**0.25**	32.17	**4.51**	**0.31**	23.68	MMACHC	c.1 A> G/c.482G> A	p.M1?/p.S525C	CH
16	CblC	**11.96**	**0.889**	8.87	**14.7**	**1.965**	7.75	MMACHC	c.609G> A/c.567dup	p.w203/p.I190Yfs*13	CH
17	CblC	**10.57**	**0.535**	12.76	**6.29**	**0.896**	**5.36**	MMACHC	c.567dup/c.609G > A	p.I190Yfs*13/p.w203*	CH
18	CblC	**8.37**	**0.691**	**6.23**	2.83	**0.21**	41.31	MMACHC	c.609G> A	p.w203*	Hom
19	CblC	**11.32**	**0.68**	12.19	**10.4**	**1.243**	12.07	MMACHC	c.609G> A	p.w203*	Hom
20	CblC	3.22	**0.404**	8.09	**4.17**	**0.263**	28.58	MMACHC	c.80 A > G/c.217 C > T	p.Q27R/p.R73*	CH
21	CblC	**4.84**	**0.258**	16.78	3.56	**0.331**	41.69	MMACHC	c.1 A> G/c.482G> A	p.G11R/p.R47*	CH
22	CblC	**7.76**	**0.566**	**5.52**	**6.17**	**0.789**	**6.67**	MMACHC	c.609G > A/c.658_660del	p.W203*/p.K220del	CH
23	CblC	**4.22**	**0.23**	22.93	NA^a^	NA^a^	NA^a^	NA^d^	NA^d^	NA^d^	NA^d^
24	CblC/TcblR	**6.15**	**1.005**	25.53	**4.15**	**0.313**	11.9	MMACHC	c.482G> A/c.80 A> G	p.R161Q/p.Q27R	CH
25	MUT	**19.02**	**0.543**	22.67	**28.47**	**0.897**	10.85	MMUT	c.2080 C > T/c.682 C>	p.R694W/p.R228X	CH
26	MUT	**32**	**0.387**	8.63	**6.69**	**0.222**	11.89	MMUT	c.1439 A> G/c.1106G> A	p.D480G/p.R369H	CH
27	MUT	**5.85**	**0.395**	24.29	**7.26**	0.148	11.79	MMUT	c.1663G> A/c.1280G> A	p.A555T/p.G427D	CH
28	MUT	**5.47**	**0.339**	45.77	**9.73**	**0.385**	43.77	MMUT	c.2150G> T/c.437 A> G	p.G717V/p.Y146C	CH
29	MUT	**19.09**	**1.258**	30.77	**9.5**	**0.936**	34.83	MMUT	c.729_730insTT/c.421G> A/c.1293G> T	p.D244Lfs*39/p.A141T/p.M4311	CH
30^c^	MUT	**15.19**	**1.208**	22.02	**8.11**	**1.438**	30.23	MMUT	c.1850T > G/c.1170del	p.L617R/p.F390Lfs*10	CH
31^c^	MUT	**15.27**	**0.669**	68.01	NA^b^	NA^b^	NA^b^	MMUT	c.755dupA/c.1214 C> T	p.H252Qfs*6/p.A405V	CH
32	MUT	**12.57**	**0.721**	29.06	**29.13**	**1.202**	11.81	MMUT	NA^d^	NA^d^	NA^d^
33	MUT/CblJ	**16.3**	**0.709**	17.66	**8.26**	**0.215**	**6.69**	MMUT /ABCD4	c.1630_1631delinsTA/c.1829G> C/c.1574 C> G	p.G544*/p.R610P/p.S525C	CH
34	TCblR	3.43	**0.305**	19.92	2.97	**0.312**	25.87	CD320	c.262_264del	p.E88del	Hom
35^b^	/	**9.88**	**0.825**	24.08	**9.38**	**0.868**	20.65	/	/	/	
36^b^	/	**10.63**	**0.448**	7.71	**4.89**	**1.164**	**3.4**	/	/	/	

a Data unavailable from external review centers

b Deceased prior to examination

c Cases of illness and death

d Genetic reports were not obtained due to parental non-consent

**Table 3:** MS/MS screening results and follow-up data

Abnormal indicators	Subtype	MS/MS Screening (Abnormal count/Total tested)		
		Primary Screening Results	Recall Review Results	Follow-up
C3	Combined	23/25	17/21	1/15
	Isolated	9/9	8/8	6/6
C3/C2	Combined	25/25	20/21	2/15
	Isolated	9/9	7/8	5/6
MET	Combined	8/25	5/21	1/14
	Isolated	2/9	1/8	1/6
HCY	Combined	18/21	6/9	7/10
	Isolated	1/5	0/1	0/2
UMA	Combined	13/14	7/8	3/16
	Isolated	5/5	1/1	3/4
MCA	Combined	10/15	3/8	4/16
	Isolated	4/5	0/1	2/3
HPA	Combined	6/16	3/8	3/16
	Isolated	3/6	1/1	2/3

### Molecular characteristics of the diagnosed patients

Our comprehensive genetic analysis of 32 methylmalonic acidemia (MMA) cases revealed distinct mutational patterns across disease-associated genes (Table 4), with *MMACHC* emerging as the most frequently affected locus (68.8% of variants). The c.609G > A (p.W203*) nonsense mutation represented a major hotspot, detected in 45.5% of *MMACHC*-positive cases either in homozygous state (4 cases) or as compound heterozygotes with frameshift variants like c.656_658del (p.219_220del). While *MMACHC* variants predominantly consisted of loss-of-function mutations (86.4% nonsense/frameshift), *MMUT* gene alterations (25% of cases) exhibited greater diversity, with 17 variants identified across 8 patients, including both 15 known pathogenic mutations and novel VUS (c.437 A > G). Notably, 62.5% of *MMUT* mutations involved previously unreported variant pairs, contrasting with the more uniform *MMACHC* mutation profile. Variants were identified in *CD320*, one case of *CD320* homozygous deletion (c.262_264del, variant of uncertain significance), this finding expand the mutational spectrum of MMA in the Chinese population, but the *CD320* c.262_264del is a Variant of Uncertain Significance requiring further functional validation. This spectrum analysis not only confirms MMACHC’s central role in Chinese MMA patients but also reveals important gene-specific differences: MMACHC-related cases follow classical autosomal recessive inheritance with hotspot mutations, whereas MMUT-associated MMA displays greater genetic heterogeneity, potentially explaining the broader phenotypic variability observed in clinical practice [15, 16].

**Table 4:** Health Follow-up status of 26 pediatric patients

No.	Gender	Age	Developmental Status
1	Female	8y8m	Overweight
3	Male	3 m	Deceased
4	Female	8y3m	Neurodevelopmental Delay
7	Female	7y9m	Growth and Developmental Delay
8	Male	7y3m	Severe Obesity, Neurodevelopmental Delay
9	Female	6y6m	Neurodevelopmental Delay
19	Male	4y7m	Severe Wasting
24	Female	3y5m	Neurodevelopmental Delay
25	Female	3y5m	Growth and Developmental Delay
30	Male	1 m	Deceased
32	Female	/	Deceased
7	Female	7y9m	Growth and Developmental Delay

### Follow-up study reveals growth and developmental characteristics in children with MMA

A longitudinal follow-up study of 26 children with methylmalonic acidemia (MMA) (3 deceased during follow-up) identified significant growth and developmental abnormalities in the 23 surviving patients. Nutritional status assessment revealed that 21.7% (5/23) of the children exhibited physical growth disorders, including 2 cases of growth stunting (height-for-age < -2SD), 1 case of severe wasting (weight-for-height < -3SD), as well as 2 overweight and 1 severely obese child. Neurological evaluations detected neurodevelopmental delays in 17.4% (4/23) of patients, including one child with an *MMAB* gene variant (age 6) who lost schooling capacity and another with a *MMUT* gene variant (age 7) who became incapable of self-care(Table 4).

Children with the CblC subtype generally respond well to treatment regimens including vitamin B12, L-carnitine, betaine, and calcium folinate. In most cases, blood levels of C3, C3/C0, C3/C2, and urinary methylmalonic acid (MAA) can be effectively reduced to near-normal ranges, with homocysteine (HCY) maintained below 30 µmol/L. In contrast, patients with MUT or CblB subtypes are managed with specialized metabolic formula, a low-protein diet, and L-carnitine, sometimes supplemented with vitamin B12. Their treatment outcomes are generally less favorable compared to the CblC subtype, showing insignificant decreases in blood C3, C3/C0, C3/C2, and urinary MAA. Among the 6 MUT-type patients followed at our center, 3 exhibited developmental delay, as did 1 CblB-type patient. Notably, one MUT-type patient (Case 27) presented only mildly elevated blood C3, C3/C0, C3/C2, and urinary MAA, yet developed severe autism and is unable to perform daily living activities independently. These results not only confirm the long-term health challenges faced by children with MMA but also emphasize the importance of establishing a multidisciplinary follow-up system, particularly for personalized monitoring strategies tailored to patients with different genotypes. The study data provide critical evidence for optimizing long-term management strategies for MMA patients. 

## Discussion

This large-scale study of 782,930 neonates screened in Hefei (2016–2023) confirms MMA as a clinically significant metabolic disorder among Chinese newborns, demonstrating an overall detection rate of 4.3 per 100,000 births. The research reveals that *MMACHC* mutations dominate the genetic architecture (68.8% of cases), with c.609G > A emerging as a distinctive regional hotspot variant. Longitudinal follow-up data further established critical genotype-phenotype correlations, with MMAB variants preferentially associated with cognitive impairment and Mut variants linked to progressive motor dysfunction. These collective findings strongly support implementing population-specific screening strategies prioritizing MMACHC c.609G > A detection, developing subtype-targeted treatment regimens, and establishing comprehensive multidisciplinary monitoring protocols to prevent neurodevelopmental complications in affected children. This study provides a comprehensive analysis of the epidemiological, metabolic, and genetic characteristics of methylmalonic acidemia (MMA) in a large Chinese neonatal cohort from Hefei, integrating findings from newborn screening (NBS), biochemical profiling, and molecular diagnostics. Our results highlight key regional distinctions in mutation spectra, metabolic signatures, and clinical outcomes, offering insights for optimizing screening protocols and personalized management strategies.

The observed MMA prevalence of 4.34 per 100,000 births in Hefei aligns with prior reports from other Chinese regions (e.g., 1/29,601 in a multicenter NGS study) but remains significantly higher than global estimates (1/50,000–1/125,000) [1]. In combined methylmalonic acidemia cases, *MMACHC* gene mutations were the most prevalent, accounting for 88% (22/25) of cases. The *MMACHC* c.609G > A (p.W203*) variant emerged as a regional hotspot (45.5% of *MMACHC* alleles), consistent with its high carrier frequency in Chinese populations [17, 18]. In contrast, *MMUT* variants exhibited greater diversity, with 62.5% being novel or rare, reflecting broader mutational heterogeneity [19]. According to research reports that functional studies of *MMUT* missense mutations (e.g., L140P, G161V) confirmed their pathogenicity through impaired enzyme stability and activity, reinforcing the need for variant-specific management [20].

The identification of rare variants in *CD320*, *MMAB*, and *MCEE* (3.1% each) expands the mutational spectrum. The *CD320* c.262_264del variant, though of uncertain significance, may represent a novel founder mutation warranting functional validation. Notably, *MMAB*-associated cases showed distinct neurocognitive deficits, while *MMUT* variants correlated with motor deterioration, echoing genotype-phenotype patterns observed in European cohorts [15].

Longitudinal follow-up revealed significant morbidity, including growth impairment (21.7%) and neurodevelopmental delays (17.4%), despite early intervention. The high mortality rate (11.5%) in our cohort—attributed to metabolic crises—parallels global data, emphasizing the need for aggressive acute management protocols [21]. The poorer outcomes in *MUT*-deficient patients (non-responsive to B12) highlight the urgency of alternative therapies, such as liver transplantation or emerging gene therapies targeting *MUT* restoration [21]. The decline in diagnostic age (54 to 43 days) reflects improved screening efficiency but remains suboptimal compared to ideal timelines (< 14 days). Integrating NGS as a first-tier test could further reduce delays, as demonstrated by Chen et al., who achieved a 70.8% positive predictive value (PPV) versus 5.3% for MS/MS alone. However, the higher false-negative rate of NGS (26.1% for MMUT variants) necessitates a combined MS/MS-NGS approach to maximize sensitivity [22, 23].

This study’s retrospective design and single-center data may limit generalizability. The absence of functional assays for variant of uncertain significance (e.g., *CD320* c.262_264del) warrants further investigation. Additionally, long-term outcomes beyond childhood remain uncharacterized; conducting long-term and scientifically rigorous extended follow-up could elucidate adult-onset complications, such as renal or cardiovascular sequelae.

Future efforts to optimize MMA management should adopt a multifaceted approach, beginning with regional screening customization that prioritizes *MMACHC* testing in high-prevalence areas while expanding *MMUT* coverage in regions with isolated-type predominance. To enhance diagnostic precision, metabolomic integration of C3/C2 ratios with methionine (Met) and homocysteine (HCY) profiling should be implemented for accurate subtyping. Concurrently, therapeutic innovation must be accelerated, including clinical trials for *MMUT*-targeted gene therapies and optimization of B12 regimens for *MMACHC* variants to address unmet needs in treatment efficacy. Finally, a paradigm shift toward multidisciplinary care is essential, incorporating standardized protocols for neurodevelopmental surveillance and nutritional support to mitigate long-term morbidity and improve quality of life for affected individuals. This comprehensive strategy aligns precision medicine with practical clinical implementation to address the heterogeneous challenges posed by MMA.

## Conclusion

Our findings delineate the biochemical and genetic landscape of MMA in Hefei, emphasizing the centrality of *MMACHC* c.609G > A and the clinical heterogeneity driven by genotype. By aligning screening strategies with regional mutation profiles and advancing genotype-specific therapies, we can mitigate the substantial morbidity and mortality associated with this disorder.

## Data Availability

The data that support the findings of this study are available from the corresponding author upon reasonable request. The data are not publicly available due to privacy or ethical restrictions.
